# Supplementary material for: Exploring the Role of Endoplasmic Reticulum Stress in Hepatocellular Carcinoma through mining of the Human Protein Atlas
Source: Biology (Basel). 2021 Jul 9;10(7):640. doi: 10.3390/biology10070640 (PMC8301178; doi:10.3390/biology10070640)
Supplement: Supplementary file 1 [file biology-10-00640-s001.zip › biology-1282419-supplementary.pdf]

biology

MDPI

*Article*

**Supplementary Table S1.** Full UPR gene set.

| Gene description | Gene name |
| --- | --- |
| Heat shock protein family A (Hsp70) member 5 | BiP/GRP78 |
| Calreticulin | CALR |
| Calnexin | CANX |
| Protein disulfide isomerase family A member 3 | PDIA3 |
| Protein disulfide isomerase family A **member** 4 | PDIA4 |
| Protein disulfide isomerase family A member 5 | PDIA5 |
| Glucosidase II alpha subunit | GANAB |
| Hypoxia up-regulated 1 | HYOU1 |
| Endoplasmic reticulum to nucleus signaling 1 | IRE1 alpha |
| Endoplasmic reticulum to nucleus signaling 2 | IRE1 beta |
| Phosphoinositide-3-kinase regulatory subunit 1 | PIK3R1 |
| X-box binding protein 1 | XBP1 |
| Mitogen-activated protein kinase kinase kinase 5 | ASK1 |
| DnaJ heat shock protein family (Hsp40) member B9 | DNAJB9 |
| ER degradation enhancing alpha-mannosidase like protein 1 | EDEM1 |
| Mitogen-activated protein kinase 8 | JNK1 |
| Mitogen-activated protein kinase kinase kinase 5 | MAP3K5 |
| TNF receptor associated factor 2 | TRAF2 |
| Presenilin 1 | PSEN1 |
| Presenilin 2 | PSEN2 |
| Receptor for activated C kinase 1 | RACK1 |
| Protein disulfide isomerase family A member 6 | PDIA6 |
| Eukaryotic translation initiation factor 2 alpha kinase 3 | PERK |
| Eukaryotic translation initiation factor 2A | EIF2A |
| Eukaryotic translation initiation factor 2 alpha kinase 1 | EIF2AK1 |
| Eukaryotic translation initiation factor 2 alpha kinase 2 | EIF2AK2 |
| Eukaryotic translation initiation factor 2 alpha kinase 4 | EIF2AK4 |
| Eukaryotic translation initiation factor 2 subunit alpha | EIF2S1 |
| Eukaryotic translation initiation factor 2 subunit beta | EIF2S2 |
| Eukaryotic translation initiation factor 2 subunit gamma | EIF2S3 |
| Activating transcription factor 3 | ATF3 |
| Activating transcription factor 4 | ATF4 |
| Tribbles pseudokinase 3 | TRIB3 |
| DNA damage inducible transcript 3 | CHOP |
| Protein phosphatase 1 regulatory subunit 15A | GADD34 |
| Protein phosphatase 1 catalytic subunit beta | PPP1CB |
| Protein phosphatase 1 catalytic subunit gamma | PPP1CC |
| Endoplasmic reticulum oxidoreductase 1 alpha | ERO1 alpha |
| Endoplasmic reticulum oxidoreductase 1 beta | ERO1 beta |
| Activating transcription factor 5 | ATF5 |
| Homocysteine inducible ER protein with ubiquitin like domain 1 | HERPud1 |
| Heat shock protein 90 beta family member 1 | GRP94/HSP90B1 |
| Nuclear factor, erythroid 2 like 1 | NFE2L1 |
| Nuclear factor, erythroid 2 like 2 | NFE2L2 |

| | |
|---|---|
| Baculoviral IAP repeat containing 2 | BIRC2 |
| Baculoviral IAP repeat containing 3 | BIRC3 |
| Unc-51 like autophagy activating kinase 1 | ULK1 |
| Lysosomal associated membrane protein 3 | LAMP3 |
| DnaJ heat shock protein family (Hsp40) member C3 | p58ipk |
| Activating transcription factor 6 | ATF6 |
| Activating transcription factor 6 beta | ATF6 beta |
| Membrane bound transcription factor peptidase, site 1 | MBTPS1 |
| Membrane bound transcription factor peptidase, site 2 | MBTPS2 |
| DnaJ heat shock protein family (Hsp40) member A3 | DNAJA3 |
| DnaJ heat shock protein family (Hsp40) member B1 | DNAJB1 |
| DnaJ heat shock protein family (Hsp40) member B2 | DNAJB2 |
| DnaJ heat shock protein family (Hsp40) member B6 | DNAJB6 |
| DnaJ heat shock protein family (Hsp40) member B9 | DNAJB9 |
| DnaJ heat shock protein family (Hsp40) member B11 | DNAJB11 |
| DnaJ heat shock protein family (Hsp40) member B12 | DNAJB12 |
| DnaJ heat shock protein family (Hsp40) member B14 | DNAJB14 |
| DnaJ heat shock protein family (Hsp40) member C1 | DNAJC1 |
| DnaJ heat shock protein family (Hsp40) member C3 | DNAJC3 |
| DnaJ heat shock protein family (Hsp40) member C5 | DNAJC5 |
| DnaJ heat shock protein family (Hsp40) member C5 beta | DNAJC5B |
| DnaJ heat shock protein family (Hsp40) member C7 | DNAJC7 |
| DnaJ heat shock protein family (Hsp40) member C10 | DNAJC10 |
| DnaJ heat shock protein family (Hsp40) member C12 | DNAJC12 |
| DnaJ heat shock protein family (Hsp40) member C21 | DNAJC21 |
| Exosome component 1 | EXOSC1 |
| Exosome component 2 | EXOSC2 |
| Exosome component 3 | EXOSC3 |
| Exosome component 4 | EXOSC4 |
| Exosome component 5 | EXOSC5 |
| Exosome component 6 | EXOSC6 |
| Exosome component 7 | EXOSC7 |
| Exosome component 8 | EXOSC8 |
| Exosome component 9 | EXOSC9 |
| Mitogen-activated protein kinase 14 | MAPK14 |
| Calumenin | CALU |
| Peptidylprolyl isomerase B | PPIB |
| Prolyl 4-hydroxylase subunit beta | P4HB |
| Asparagine synthetase (glutamine-hydrolyzing) | ASNS |
| Protein phosphatase 2 regulatory subunit B'beta | PPP2R5B |
| C-C motif chemokine ligand 2 | CCL2 |
| WD repeat domain, phosphoinositide interacting 1 | WIP1 |
| KDEL endoplasmic reticulum protein retention receptor 3 | KDELR3 |
| SHC adaptor protein 1 | SHC1 |
| Tripeptidyl peptidase 1 | TPP1 |
| Heparin binding growth factor | HDFG |
| Talin 1 | TLN1 |
| Exostosin like glycosyltransferase 3 | EXTL3 |
| TSPY like 2 | TSPYL2 |

| | |
|---|---|
| Dynactin subunit 1 | DCTN1 |
| Sulfotransferase family 1A member 4 | SULT1A4 |
| Poly(A)-specific ribonuclease | PARN |
| Adducin 1 | ADD1 |
| Zinc finger and BTB domain containing 17 | ZBTB17 |
| Acyl-CoA dehydrogenase very long chain | ACADVL |
| SEC31 homolog A, COPII coat complex component | SEC31A |
| Signal sequence receptor subunit 1 | SSR1 |
| Signal sequence receptor subunit 2 | SSR2 |
| ADP ribosylation factor GTPase activating protein 1 | ARFGAP1 |
| Prolactin regulatory element binding | PREB |
| ATPase H+ transporting V0 subunit d1 | ATP6V0D1 |
| Lamin A/C | LMNA |
| FK506 binding protein 14 | FKBP14 |
| Stress associated endoplasmic reticulum protein 1 | SERP1 |
| SRP receptor beta subunit | SRPRB |
| CTD small phosphatase 2 | CTDSP2 |
| Golgi SNAP receptor complex member 2 | GOSR2 |
| Yip1 interacting factor homolog A, membrane trafficking protein | YIF1A |
| DEAD/H-box helicase 11 | DDX11 |
| Wolframin ER transmembrane glycoprotein | WFS1 |
| Synoviolin 1 | SYVN1 |
| Kelch domain containing 3 | KLHDC3 |
| DIS3 homolog, exosome endoribonuclease and 3'-5' exoribonuclease | DIS3 |
| CXXC finger protein 1 | CXXC1 |
| KH-type splicing regulatory protein | KHSRP |
| Cullin 7 | CUL7 |
| Insulin like growth factor binding protein 1 | IGFBP1 |
| Glycogen synthase kinase 3 alpha | GSK3A |
| Nuclear transcription factor Y subunit beta | NFYB |
| TatD DNase domain containing 2 | TATDN2 |
| Decapping mRNA 2 | DCP2 |
| Polycystin 2, transient receptor potential cation channel | PKD2 |
| Lectin, mannose binding 1 | LMAN1 |
| Endoplasmic reticulum protein 29 | ERP29 |
| Thioredoxin domain containing 5 | TXNDC5 |

**Supplementary Table S2.** UPR prognostic markers with protein functions.

| Gene symbol | Full gene name | Function of protein product (The Human Protein Atlas) |
|---|---|---|
| RACK1 | Receptor for activated C kinase 1 | Scaffolding protein involved in the recruitment, assembly and/or regulation of a variety of signaling molecules. |
| ATF4 | Activating transcription factor 4 | Core effector required for adaptation to ER stress |
| GANAB | Glucosidase II alpha subunit | Plays a role in protein folding and quality control by cleaving glucose residues from immature glycoproteins in the ER |
| ERP29 | Endoplasmic reticulum protein 29 | Plays an important role in the processing of secretory proteins within the ER, possibly by participating in the folding of proteins in the ER |
| TPP1 | Tripeptidyl peptidase 1 | Lysosomal serine protease with tripeptidyl-peptidase I activity |
| PDIA6 | Protein disulfide isomerase family A member 6 | May function as a chaperone that inhibits aggregation of misfolded proteins |
| SSR2 | Signal sequence receptor subunit 2 | Part of a complex whose function is to bind calcium to the ER membrane and thereby regulate the retention of ER resident proteins. |
| EIF2S3 | Eukaryotic translation initiation factor 2 subunit gamma | As a subunit of eIF-2, involved in the early steps of protein synthesis. |
| EIF2AK1 | Eukaryotic translation initiation factor 2 alpha kinase 1 | Metabolic-stress sensing protein kinase that phosphorylates the alpha subunit of eIF-2-alpha in response to various stress conditions |
| EIF2S2 | Eukaryotic translation initiation factor 2 subunit beta | Initiation factor in the early steps of protein synthesis |
| CHOP | DNA damage inducible transcript 3 | Multifunctional transcription factor in ER-stress response, regulating apoptosis, cell cycle/growth arrest, transcription, UPR |
| TRIB3 | Tribbles pseudokinase 3 | Inactive protein kinase which acts as a regulator of adaptation to various stress; Inhibits the transcriptional activity of DDIT3/CHOP and is involved in DDIT3/CHOP-dependent cell death during ER stress |
| SRPRB | SRP receptor beta subunit | Ensures, in conjunction with the signal recognition particle, the correct targeting of the nascent secretory proteins to the endoplasmic reticulum membrane system |
| DNAJC1 | | Binds the molecular chaperone BiP, may modulate protein synthesis. |

| | | |
|---|---|---|
| | DnaJ heat shock protein family (Hsp40) member C1 | |
| CALU | Calumenin | Involved in ER functions such as protein folding and sorting. |
| PPP1CB | Protein phosphatase 1 catalytic subunit beta | Essential for cell division, it participates in the regulation of glycogen metabolism, muscle contractility and protein synthesis. |
| EIF2A | Eukaryotic translation initiation factor 2A | Functions in the early steps of protein synthesis of a small number of specific mRNAs |
| SSR1 | Signal sequence receptor subunit 1 | Binds calcium to the ER membrane and thereby regulate the retention of ER resident proteins. May function as a membrane-bound chaperone facilitating folding of translocated proteins. |
| SEC31A | SEC31 homolog A, COPII coat complex component | Promotes the formation of transport vesicles from the ER |
| KDELR3 | KDEL endoplasmic reticulum protein retention receptor 3 | Present on ER resident proteins and that mediates their recycling from the Golgi back to the ER. |
| GADD34 | Protein phosphatase 1 regulatory subunit 15A | Recruits PP1 to dephosphorylate eIF-2A/EIF2S1, thereby reversing the shut-off of protein synthesis initiated by stress-inducible kinases and facilitating recovery of cells from stress. May promote apoptosis. |
| PPP1CC | Protein phosphatase 1 catalytic subunit gamma | Essential for cell division, and participates in the regulation of glycogen metabolism, muscle contractility and protein synthesis. |
| GSK3A | Glycogen synthase kinase 3 alpha | Acts as a negative regulator in the hormonal control of glucose homeostasis, Wnt signaling and regulation of transcription factors and microtubules |
| DNAJC5 | DnaJ heat shock protein family (Hsp40) member C5 | General chaperone in regulated exocytosis |
| PIK3R1 | Phosphoinositide-3-kinase regulatory subunit 1 | Modulates the cellular response to ER stress by promoting nuclear translocation of XBP1 isoform 2 in a ER stress- and/or insulin-dependent manner during metabolic overloading in the liver and hence plays a role in glucose tolerance improvement |
| ERO1 alpha | Endoplasmic reticulum oxidoreductase 1 alpha | Involved in disulfide bond formation in the ER. Important role in ER stress-induced, CHOP-dependent apoptosis by activating the inositol 1,4,5-trisphosphate receptor IP3R1. |

| | | |
|---|---|---|
| EIF2S1 | Eukaryotic translation initiation factor 2 subunit alpha | Functions in the early steps of protein synthesis by forming a ternary complex with GTP and initiator tRNA |
| CXXC1 | CXXC finger protein 1 | Transcriptional activator that exhibits a unique DNA binding specificity for CpG unmethylated motifs. |
| TRAF2 | TNF receptor associated factor 2 | Regulates activation of NF-kappa-B and JNK and plays a central role in the regulation of cell survival and apoptosis. |
| DNAJC7 | DnaJ heat shock protein family (Hsp40) member C7 | Acts as co-chaperone regulating the molecular chaperones HSP70 and HSP90 in folding of steroid receptors, such as the glucocorticoid receptor and the progesterone receptor. |
| DNAJC21 | DnaJ heat shock protein family (Hsp40) member C21 | May act as a co-chaperone for HSP70. |
| PSEN1 | Presenilin 1 | Plays a role in Notch and Wnt signaling cascades and regulation of downstream processes via its role in processing key regulatory proteins. |
| ZBTB17 | Zinc finger and BTB domain containing 17 | Can function as an activator or repressor depending on its binding partners, and by targeting negative regulators of cell cycle progression. |
| PPP2R5B | Protein phosphatase 2 regulatory subunit B'beta | Modulates substrate specificity, subcellular localization, and responsiveness to phosphorylation. |
| EXOSC3 | Exosome component 3 | Participates in a multitude of cellular RNA processing and degradation events. |
| EXOSC9 | Exosome component 9 | |
| EXOSC2 | Exosome component 2 | |
| GOSR2 | Golgi SNAP receptor complex member 2 | Involved in transport of proteins from the cis/medial-Golgi to the trans-Golgi network. |
| DNAJB6 | DnaJ heat shock protein family (Hsp40) member B6 | Acts as an endogenous molecular chaperone for neuronal proteins |
| JNK1 | Mitogen-activated protein kinase 8 | Involved in various processes such as cell proliferation, differentiation, migration, transformation and programmed cell death. |
| DCP2 | Decapping mRNA 2 | Catalyzes the cleavage of the cap structure on mRNAs |
| ASNS | Asparagine synthetase (glutamine-hydrolyzing) | Amino-acid biosynthesis, Asparagine biosynthesis |
| EXTL3 | Exostosin like glycosyltransferase 3 | Regulates the biosynthesis of heparan sulfate (HS). Important for both skeletal development and hematopoiesis, through the formation of HS proteoglycans |
| FKBP14 | FK506 binding protein 14 | Accelerates the folding of proteins during protein synthesis. |

| | | |
|---|---|---|
| DDX11 | DEAD/H-box helicase 11 | Participates in various functions in genomic stability, including DNA replication, DNA repair and heterochromatin organization as well as in ribosomal RNA synthesis |
